# Predicting colorectal cancer survival by combined c-reactive protein and tumor immune score

**DOI:** 10.1038/s41698-025-01192-1

**Published:** 2025-11-25

**Authors:** Tafirenyika Gwenzi, Durgesh Wankhede, Tanwei Yuan, Megha Bhardwaj, Petra Schrotz-King, Sophie C. Anker, Ben Schöttker, Michael Hoffmeister, Hermann Brenner

**Affiliations:** 1 Division of Clinical Epidemiology of Early Cancer Detection, German Cancer Research Center (DKFZ) Heidelberg, Im Neuenheimer Feld 581, 69120 Heidelberg, Germany; 2 Medical Faculty Heidelberg, Heidelberg University, Im Neuenheimer Feld 672, 69120 Heidelberg, Germany; 3 Division of Primary Cancer Prevention, German Cancer Research Center (DKFZ) Heidelberg, and National Center for Tumor Diseases (NCT), NCT Heidelberg, a partnership between DKFZ and University Hospital Heidelberg, Im Neuenheimer Feld 460, 69120 Heidelberg, Germany; 4 Department of Internal Medicine and Clinical Chemistry, University Hospital Heidelberg, Im Neuenheimer Feld 400, 69120 Heidelberg, Germany; 5 Network Aging Research, Heidelberg University, Bergheimer Straße 20, 69115 Heidelberg, Germany; 6 German Cancer Consortium (DKTK), German Cancer Research Center (DKFZ), Im Neuenheimer Feld 280, 69120 Heidelberg, Germany

**Keywords:** Biomarkers, Cancer, Gastroenterology, Immunology, Oncology

## Abstract

We evaluated the joint relationship of post-operative C-reactive protein (poCRP) and a tumor immune-cell-score (IS) with overall survival (OS) and CRC-specific survival (CSS) in 680 colorectal cancer (CRC) patients recruited in Germany. CRP was assessed post-surgery while IS was derived from CD3 + /CD8+ cell densities in tumor tissue. Patients were categorized into four **C**-**R**eactive protein-**I**mmune cell **S**core (CRIS) groups: CRIS-1 (CRP-low/IS-high), CRIS-2 (CRP-low/IS-low), CRIS-3 (CRP-high/IS-high), and CRIS-4 (CRP-high/IS-low). Associations of CRIS with survival were assessed using Cox regression, and quantified by hazard ratios with 95% confidence intervals (HR, 95%CI). Subgroup analysis by presence of non-metastatic disease and time of blood draw in relation to adjuvant chemotherapy were conducted. After a median follow-up of 9.6 (IQR, 4.6–14.6) years, 214 (31.5%) patients died, 140 (20.6%) from CRC. Patients in CRIS-4 category had worse prognosis compared to CRIS-1 category [HR_(95%CI)_: 2.01 (1.32–3.08) and 2.60 (1.57–4.32) for OS and CSS, respectively]. These associations were stronger for non-metastatic disease (OS_HR_ = 2.45, CSS_HR_ = 4.49), as well as for patients with blood collected after adjuvant chemotherapy (OS_HR_ = 4.17, CSS_HR_ = 6.62). Integrating post-operative systemic inflammation and tumor immune characteristics may improve prognostic stratification of patients receiving adjuvant chemotherapy for non-metastatic CRC.

## Introduction

Colorectal cancer (CRC) is one of the leading causes of cancer-related morbidity and mortality globally^1^. Despite the pivotal role of cancer stage at diagnosis in determining long-term clinical outcomes, significant variability in survival outcomes among patients remains unexplained^2,3^. Consequently, there is a growing recognition of the need to identify additional prognostic markers that either complement or go beyond traditional staging. In this context, the systemic inflammatory response and local immune dynamics within the tumor microenvironment (TME) are emerging as critical factors in predicting survival and treatment response^4–6^, with potential to enhance clinical care.

The complex relationship between the immune system and CRC development is crucial but not fully understood, particularly with regard to how systemic and TME immune responses interact with CRC progression. Systemic inflammation, as measured by indicators such as C-reactive protein (CRP), is not only associated with tumor growth and higher malignant grades^7,8^, but also long-term survival of CRC patients^9^. In addition, the Immunoscore®, a metric that quantifies immune cell infiltration (particularly CD3+ and CD8+ cells) within tumors^10–12^, provides a more comprehensive characterization of the TME and highlights the significant prognostic value of tumor-infiltrating lymphocytes (TILs), regardless of cancer stage at diagnosis^13,14^.

Previous research which has linked systemic inflammation to CRC prognosis primarily relied on white blood cell counts to quantify systemic inflammation^5,6^. However, CRP is a highly sensitive marker of systemic inflammation, and a cost-effective alternative for assessing post-operative systemic inflammation in routine clinical practice^15^. CRP levels are highly elevated shortly after CRC surgery, mostly returning to “normal values” within two weeks to one month after surgery, after which they are strong predictors of long-term outcomes^16^. To date, the combined prognostic value of postoperative CRP (poCRP) and the Immunoscore® has not been investigated. In this study, we aimed to describe the independent and joint prognostic value of poCRP and a tumor immune cell score (IS), which is closely related to the Immunoscore®, for long-term survival outcomes in a large cohort of CRC patients.

## Results

### Patient characteristics

After a median follow-up of 9.6 years among the 680 CRC patients, 31.5% had died, with 20.6% dying from CRC; relapse occurred in 30.1%. The median age at diagnosis was 70 years, with a male predominance (61.3%), reflective of higher prevalence of CRC among males than females in the general population (Table 1). Most were diagnosed at stage II (39.6%) or stage III (32.4%), and 12.7% had stage IV disease. Over 80% had blood samples collected within a year post-surgery (median 5.3 months). Median poCRP was slightly higher in samples collected within 2 weeks—3 months, but IS remained consistent across time points (Supp. Fig. 2). poCRP and IS were neither correlated in the overall patient population nor in the subgroups by time of blood sampling or by stage at diagnosis (Supp. Fig. 3–5 and Supp. Table 1). CRIS subtyping showed that CRIS-1 to 3 was largely dominated by non-metastatic patients (stage I—III), while CRIS-4 predominated in stage IV patients (Supp. Fig. 6).

**Table 1:** Patient characteristics at baseline

Characteristic	No. (%)
Age (Median, IQR), years	70 (63–77)
<70 years	344 (50.6%)
≥70 years	336 (49.4%)
Sex	
Female	263 (36.7%)
Male	417 (61.3%)
TNM Stage	
I	105 (15.4%)
II	269 (39.6%)
III	220 (32.4%)
IV	86 (12.7%)
Cancer Site	
Colon	489 (71.9%)
Rectum	191 (28.1%)
CRC Sidedness^a^	
Left	401 (58.9%)
Right	279 (41.1%)
C-reactive Protein (Median, IQR), *mg/L*	3.8 (1.4–10.8)
Immune Cell Score (Median, IQR), *mean percentile*	50.0 (28.0–72.4)
Blood Collection Time (Median, IQR), *months*	5.3 (1.4–9.6)
2 weeks–3 months	244 (35.9%)
3–6 months	126 (18.5%)
6–12 months	201 (29.6%)
>12 months	109 (16.0%)
Body Mass Index (Median, IQR), *kg/m*^2^	26.0 (23.3–28.7)
Underweight (<18.5)	12 (1.8%)
Normal (18.5–24.9)	272 (40.0%)
Overweight (25.0–29.9)	274 (40.3%)
Obese (≥30.0)	122 (17.9%)
Adjuvant Chemotherapy^a^	
Yes	296 (43.5%)
Death Events	214 (31.5%)
CRC Deaths	140 (20.6%)
CRC Relapse Events	205 (30.1%)
Follow-up (Median, IQR), *years*	9.6 (4.6–14.6)

^a^Missingness in variables: CRC sidedness (3.24%, *n* = *22*); Adjuvant chemotherapy (0.29%, *n* = *2*).

*CRC* colorectal cancer, *IQR* interquartile range, *TNM* tumor-node-metastasis.

### Individual associations of post-operative CRP and immune cell score with survival outcomes

Dose-response analyses revealed consistent associations of both poCRP and IS with survival across the three outcomes (Supp. Fig. 7). Higher poCRP levels were strongly linked to worse survival, while higher IS correlated with better survival outcomes. Multivariable Cox regression confirmed these associations after adjusting for key prognostic variables (Table 2). However, for the association between individual poCRP and survival stratified by time of blood collection, poCRP measured 3–6 months after surgery consistently showed no significant associations with survival outcomes (Supp. Table 2).

**Table 2:** Cox regression for the individual and mutually adjusted associations of post-operative C-reactive protein and the Immune cell score with survival outcomes

Prognostic Outcome		C-reactive protein (HR, 95% CI)			Immune cell score (HR, 95% CI)	
	Category (event rate)	Model 1	Model 2	Category (event rate)	Model 1	Model 2
Overall Survival	Low (22.2%)	1.00 (Ref)	1.00 (Ref)	High (26.1%)	1.00 (Ref)	1.00 (Ref)
	High (40.8%)	**2.09 (1.51–2.90)**	**2.00 (1.44–2.79)**	Low **(**41.1%)	1.30 **(**0.89–1.89)	1.27 **(**0.88–1.84)
	per SD increase	**1.14 (1.04–1.26)**	**1.13 (1.03–1.25)**	per SD decrease	**1.17 (1.01–1.35)**	**1.16 (1.00–1.34)**
	*P*_*interaction*_ = *0.318*					
CRC Specific Survival^a^	Low (12.8%)	1.00 **(**Ref)	1.00 **(**Ref)	High **(**15.6%)	1.00 **(**Ref)	1.00 **(**Ref)
	High (28.9%)	**2.52 (1.67–3.81)**	**2.36 (1.55–3.58)**	Low **(**31.8%)	1.41 **(**0.90–2.20)	1.35 **(**0.87–2.11)
	per SD increase	**1.17 (1.06–1.30)**	**1.16 (1.05–1.28)**	per SD decrease	**1.27 (1.05–1.53)**	**1.25 (1.04–1.51)**
	*P*_*interaction*_ = *0.455*					
Relapse-free Survival^a^	Low (22.7%)	1.00 (Ref)	1.00 (Ref)	High (24.1%)	1.00 (Ref)	1.00 (Ref)
	High (35.3%)	**1.65 (1.18–2.31)**	**1.59 (1.13–2.22)**	Low (41.5%)	1.38 (0.95–2.00)	1.37 (0.94–1.99)
	per SD increase	**1.14 (1.02–1.27)**	**1.14 (1.02–1.26)**	per SD decrease	**1.27 (1.08–1.49)**	**1.26 (1.08–1.47)**
	*P*_*interaction*_ = *0.394*					

^a^Competing risk models were applied accounting for non-CRC deaths as a competing risk using the Fine-Gray model.

Model 1 is adjusted for age + sex + TNM stage + body mass index + tumor site + tumor sidedness + adjuvant chemotherapy + time of blood sampling.

Model 2 is adjusted for Model 1 covariates + C-reactive protein/Immune cell score mutual adjustment.

*P*_*interaction*_ was obtained by modeling a continuous-by-continuous interaction model (CRP × IS).

Bold numbers show statistically significant associations.

*HR* hazard ratio, *Ref* reference, *SD* standard deviation, *TNM* tumor-node-metastasis.

### Associations of the CRIS profile with survival outcomes

**Fig. 1:**
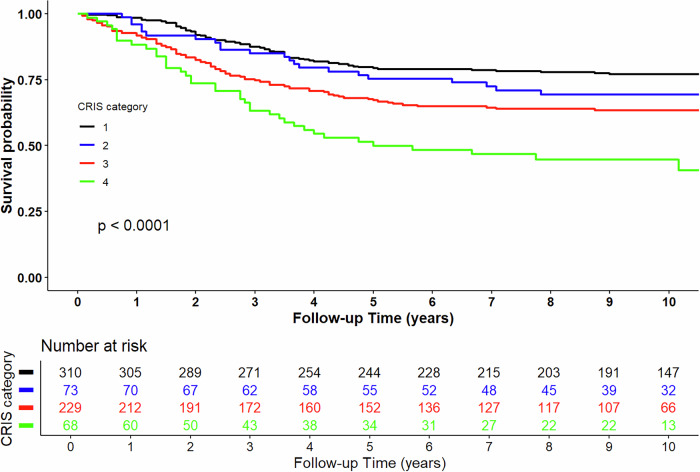
Kaplan-Meier plots for the overall survival of patients by the CRIS profile.

Patients classified as CRIS-4 had the poorest OS compared to those in all the other CRIS categories (Fig. 1), with similar trends observed for CSS and RFS (Supp. Fig. 8). Multivariable Cox regression confirmed significantly worse outcomes for CRIS-4 patients versus CRIS-1 [HR, 95%CI: 2.01 (1.32–3.08), 2.60 (1.57–4.32), and 2.08 (1.35–3.19) for OS, CSS, and RFS, respectively] (Table 3). Moreover, adding the CRIS profile to prognostic models that included age and TNM stage significantly improved 10 year overall model discrimination and calibration, as shown by increased C-indices and lower Integrated Brier Scores (Supp. Table 3). Subgroup analyses showed even stronger associations in non-metastatic (stage I–III) compared to late-stage patients (Supp. Table 4). Sensitivity analyses limited to patients with blood samples collected 2 weeks—3 months post-surgery showed significant exposure-outcome associations only for RFS (Supp. Table 5).

**Table 3:** Cox regression analysis for the associations of CRIS profile with survival outcomes

Prognostic Outcome	CRIS category	e/n (event rate)	Model 1 (HR, 95% CI)	Model 2 (HR, 95% CI)	P-trend
Overall Survival	1	70/310 **(**22.6%)	1.00 **(**ref)	1.00 **(**ref)	*<0.001*
	2	22/73 **(**30.1%)	1.37 **(**0.85–2.21)	1.18 **(**0.72–1.94)	
	3	83/229 **(**36.2%)	**1.85 (1.35–2.54)**	**1.92 (1.39–2.66)**	
	4	37/68 **(**54.4%)	**3.23 (2.17–4.79)**	**2.01 (1.32–3.08)**	
CRC Specific Survival^a^	1	39/310 **(**12.6%)	1.00 **(**ref)	1.00 **(**ref)	*<0.001*
	2	16/73 **(**21.9%)	**1.79 (1.00–3.20)**	1.50 **(**0.81–2.76)	
	3	54/229 **(**23.6%)	**2.11 (1.40–3.19)**	**2.47 (1.61–3.77)**	
	4	31/68 **(**45.6%)	**4.56 (2.84–7.31)**	**2.60 (1.57–4.32)**	
Relapse-Free Survival^a^	1	68/310 **(**21.9%)	1.00 **(**ref)	1.00 **(**ref)	*<0.001*
	2	25/73 **(**34.2%)	**1.64 (1.04–2.60)**	1.48 **(**0.92–2.38)	
	3	70/229 **(**30.6%)	**1.65 (1.17–2.28)**	**1.77 (1.26–2.48)**	
	4	36/68 **(**52.9%)	**3.61 (2.41–5.39)**	**2.08 (1.35–3.19)**	

^a^Models for CSS and RFS predictions accounted for non-CRC death as a competing risk using the Fine-Gray model

Multivariable model was adjusted for sex + age + TNM stage + BMI + cancer site + cancer sidedness + adjuvant chemotherapy + time of blood sampling; Bold numbers show statistically significant associations. P-trend was based on modeling CRIS as a numeric variable.

*CI* confidence interval, *CRC* colorectal cancer, *e/n* events/total participants, *HR* hazard ratio, *ref* reference.

### Secondary analysis

Supp Figure 8 illustrates the distribution of patients by post-operative blood sampling time relative to receipt of adjuvant chemotherapy. Most chemotherapy recipients had stage III CRC. Patients sampled before chemotherapy showed elevated poCRP levels, likely due to proximity to surgery. The distribution of IS was similar for all patients regardless of the time of blood sampling relative to receipt of chemotherapy. Among those sampled after completing chemotherapy, the CRIS-4 category was significantly associated with worse prognosis compared to CRIS-1, with hazard ratios of 4.17, 6.62, and 3.82 for OS, CSS, and RFS, respectively (Table 4). These associations were absent for patients with blood sampled before or during chemotherapy.

**Table 4:** Analysis by time of blood draw in relation to receipt of adjuvant chemotherapy

Outcome	CRIS category	Before Chemotherapy (*n* = 184)^1^	During Chemotherapy (*n* = 103)^2^	After Chemotherapy (*n* = 118)^3^
		HR (95%CI)	HR (95%CI)	HR (95%CI)
Overall Survival	1	1.00 (ref)	1.00 (ref)	1.00 **(**ref)
	2	1.05 (0.24–4.51)	1.15 (0.31–4.35)	2.13 **(**0.76–5.99)
	3	1.45 (0.58–3.67)	1.42 (0.64–3.17)	**3.27 (1.27–8.41)**
	4	1.88 (0.70–5.01)	2.02 (0.63–6.49)	**4.17 (1.39–12.50)**
CRC-Specific Survival^a^	1	1.00 (ref)	1.00 (ref)	1.00 **(**ref)
	2	1.19 (0.26–5.01)	3.05 (0.72–12.98)	2.40 **(**0.67–8.54)
	3	1.49 (0.53–4.21)	1.85 (0.72–4.79)	**5.25 (1.68–16.38)**
	4	1.65 (0.54–5.08)	2.27 (0.64–8.04)	**6.62 (1.84–23.79)**
Relapse-Free Survival^a^	1	1.00 (ref)	1.00 (ref)	1.00 **(**ref)
	2	0.76 (0.20–2.90)	2.21 (0.66–7.34)	**2.67 (1.06–6.73)**
	3	0.93 (0.45–1.90)	1.80 (0.81–3.99)	**3.42 (1.48–7.93)**
	4	1.50 (0.69–3.28)	1.82 (0.64–5.16)	**3.82 (1.37–10.72)**

^a^Models accounted for non-CRC death as a competing risk using the Fine-Gray model.

^1^Events were 75, 60 and 85 for all-cause deaths, CRC-specific deaths, and disease relapse, respectively.

^2^Events were 35, 28 and 39 for all-cause deaths, CRC-specific deaths, and disease relapse, respectively.

^3^Events were 32, 25 and 39 for all-cause deaths, CRC-specific deaths, and disease relapse, respectively.

Multivariable model was adjusted for age + sex + TNM stage + BMI.

*CI* confidence interval, *CRC* colorectal cancer, *HR* hazard ratio, *ref* reference.

## Discussion

We developed and evaluated a novel prognostic CRIS profile, combining post-operative systemic inflammation with local immune response in a large population-based cohort of CRC patients undergoing surgery. The CRIS profile was strongly predictive of OS, CSS and RFS outcomes compared to either poCRP or IS alone. The combination of poCRP-high/IS-low was independently associated with significantly worse survival outcomes. These associations were more pronounced in patients with non-metastatic CRC, as well as in patients for which the CRIS profile was assessed after the completion of adjuvant chemotherapy.

Systemic inflammation is a critical factor consistently associated with CRC progression, advanced stage, higher tumor grade, and adverse CRC outcomes^7,8^. Our previous work showed that poCRP, is a strong independent prognostic marker for long-term survival outcomes in CRC patients^9,16^. On the other hand, high TILs, particularly CD3+ and CD8 + T cells, are associated with improved response to therapy and survival, independent of cancer stage^12^. Our research group has also validated and summarized the individual and joint prognostic value of TILs in previous studies^14,17^.

Elevated poCRP levels beyond two weeks after surgery may reflect persistent systemic inflammation, possibly due to residual tumor burden, micro-metastatic disease, or complications from surgery^18^. Consequently, elevated systemic inflammation (as measured by poCRP) is associated with worse health outcomes, including worse long-term survival^9^. The observed non-significant associations between poCRP levels assessed 3–6 months and survival outcomes may suggest the role of adjuvant chemotherapy (typically given within 6 months) in altering inflammatory markers, temporarily masking a true association. IS reflects the local immune response to the tumor, which is a reliable and reproducible indicator of the host’s immune response against tumor cells, thus influencing tumor recurrence^19^. Numerous large studies also confirm that a high IS, indicating extensive lymphoid infiltration, is significantly associated with improved prognosis and survival in all stages of colon cancer^20–22^. The progressive worsening of outcomes from CRIS-1 through CRIS-4 in our findings likely reflects an interplay between systemic and local immune responses—patients with both low systemic inflammation and high intratumoral T-cell infiltration (CRIS-1) mount a coordinated antitumor defense, whereas those with systemic inflammation and suppressed local immunity (CRIS-4) exhibit an immune-evasive, tumor-promoting environment. Intermediate profiles (CRIS-2 and CRIS-3) represent partial activation or suppression of these axes, going along with proportionate survival gradients, largely driven by systemic inflammation.

Adding the CRIS profile to the prognostic model with age and TNM stage improved the overall prognostic performance for predicting OS, CSS and RFS. A recent study showed longer disease-free survival of stage II and III colon cancer patients with low pre-operative blood cell count based systemic inflammation (as quantified by lymphocytes and neutrophils) combined with high CD8 + ^6^. Similarly, stage II colon cancer patients with high pre-operative white blood cell count and low intratumoral chronic inflammatory cell density (as measured by lymphocytes and macrophages) had worse OS and RFS rates^4^. However, these studies had more limited sample sizes and relied on pre-operative white blood cell counts for the quantification of systemic inflammation. Our results expand preliminary evidence by demonstrating the value of monitoring the post-operative systemic inflammatory response in combination with IS for long-term prognostication, particularly in non-metastatic CRC patients. Implementation in clinical routine would be straightforward, as CRP is a highly sensitive marker of systemic inflammation, cost-effective, and routinely available in many clinical settings^15^.

We observed stronger predictor-outcome associations in non-metastatic (stage I–III) compared to late-stage patients (stage IV). In early disease, outcomes are driven by tumor–immune balance and peri-operative biology, both of which are captured by poCRP (systemic inflammation) and immune response within the TME^23^. In contrast, survival in metastatic CRC is dominated by tumor burden, lines of systemic therapy, and treatment heterogeneity, which can dilute biomarker influence on prognosis. Among patients with blood samples collected 2 weeks–3 months post-surgery, significant exposure-outcome associations were only seen for RFS. Peri-operative inflammation and transient immunosuppression can promote seeding and early recurrence, possibly making CRIS more predictive for RFS, a more proximal endpoint, than for downstream endpoints such as OS or CSS^22^.

The prognostic value of the CRIS profile was most pronounced when blood samples were collected after chemotherapy completion, suggesting a potential interaction between immune-inflammatory response and treatment timing. This temporal pattern suggests that CRP assessment in the period after adjuvant chemotherapy may more accurately reflect the long-term systemic inflammatory response or residual disease burden, as opposed to treatment-induced inflammation during chemotherapy^24,25^. Moreover, immune cell infiltration, being a stable histological marker, retains its prognostic significance regardless of sampling time, reinforcing the robustness of the CRIS profile for patient risk-stratification. The null associations seen in patients assessed during adjuvant chemotherapy may reflect the transient immuno-inflammatory side-effects induced by chemotherapy, which can both suppress immune responses and cause tissue inflammation, thus masking prognostic signals^26^. The lack of association in the “pre-chemotherapy” group may suggest that CRP in the immediate post-operative period is less informative due to its sensitivity to surgical stress and acute phase responses^9,16,27^.

While CRP and immune infiltration have each been studied independently, their integration into the CRIS profile offers a pragmatic and biologically meaningful tool for post-operative risk stratification. Patients classified as CRIS-4 may benefit from closer surveillance, extended adjuvant therapy, or enrollment into trials investigating anti-inflammatory or immunomodulatory strategies.

However, several limitations must be acknowledged, including the fact that some subgroup analyses were limited by small case numbers despite the large size of our patient cohort. Our study utilized single time point CRP measurements, with the timing of CRP measurements after surgery varying considerably, often occurring weeks to months after surgery. Finally, our analysis did not account for post-operative complications. However, emerging evidence shows that poCRP is a robust predictor of long-term survival outcomes regardless of its potential origins^28^.

Future studies should validate our findings, as well as establish the optimal time and cut-off for poCRP assessment in non-metastatic CRC patients receiving adjuvant therapy. In addition, the prognostic value should be evaluated in comparison and in combination with other established liquid biopsy-based prognostic biomarkers such as circulating tumor DNA and cell-free DNA.

In conclusion, the CRIS profile represents a novel and clinically relevant prognostic classifier in CRC, with the potential to guide personalized management strategies. Our findings reinforce the importance of both local immune responses and systemic inflammation in shaping CRC outcomes, particularly in the post-treatment setting. Future prospective validation and integration into clinical workflows could facilitate the implementation of CRIS in routine oncology practice.

## Methods

### Study design and study participants

Our analysis uses data from the DACHS study, a large-scale prospective population-based case-control study from Germany with additional comprehensive follow-up of CRC cases. Full details of the study design, participant recruitment, data collection, and follow-up methods have been previously reported elsewhere^29^. Briefly, patients aged 30 years and older who were newly diagnosed with CRC were recruited in more than 20 clinics in the Rhine-Neckar region of Germany between 2003 and 2021. Comprehensive sociodemographic, lifestyle, and medical data were obtained in face-to-face interviews using standardized questionnaires either during first hospitalization for CRC surgery or in the following weeks to months after discharge. Following the interviews, whole blood samples were collected, centrifuged at 1700 g for 10 minutes, and serum was stored at -80 °C for subsequent analyses. Medical details regarding tumor stage, location, and treatment were obtained from hospital records. Mortality data were ascertained by linkage to population registries, and causes of death were verified by death certificates according to International Statistical Classification of Diseases, 10^th^ Revision (ICD-10) codes C18–C20 for CRC-specific mortality. The study adheres to the tenets of the Declaration of Helsinki and was approved by the state medical boards of Baden-Württemberg and Rhineland-Palatinate, and the ethics committee of the medical faculty of Heidelberg University (ethical code: 310/2001 approved on 6 December 2001). Written informed consent was obtained from each participant.

Supplementary Figure 1 shows the patient selection criteria for our current analysis, which focused on 1143 CRC patients recruited between 2003 and 2010 for whom post-operative blood samples, tumor tissue immune cell characterization and survival follow-up data through December 2021 were available. Patients who experienced CRC relapse prior to blood draw, as well as those who had incomplete resection status (R1/R2, stage I–III) were excluded. Moreover, to avoid the confounding effects of treatment on tumor characteristics, all patients who received neoadjuvant therapy were excluded. In the primary analysis, we excluded patients with blood samples taken within two weeks post-surgery, as it is well-established that systemic inflammation is extremely variable and strongly influenced by the direct effects of surgery, acute care interventions, or potential complications in the immediate post-surgical period^9,16,27^. For the secondary exploratory analysis, we included only those patients who had receive adjuvant chemotherapy.

### Laboratory measurement of post-operative serum C-reactive protein

We measured poCRP levels in patient sera using immunoturbidimetric assays on a Siemens Healthineers ADVIA XPT system (Siemens Healthineers AG, Germany) with a lower sensitivity threshold of 2.0 mg/L. Values below the limit of detection were replaced by the lower sensitivity threshold value (*n* = 40). The interassay coefficient of variation (CV) of the system, as provided by the manufacturer, remained below 10%. For statistical analysis, poCRP levels were categorized into two groups derived from a validated optimal cut-off value of 5 mg/L based on our previous publication utilizing the DACHS and UK Biobank studies as follows: CRP-low (<5.0 mg/L) and CRP-high (≥5.0 mg/L)^16^. All laboratory assays, including CRP measurements and immune cell quantification, were conducted by trained laboratory personnel who were blinded to patient clinical characteristics and survival outcomes.

### Determination of the immune cell score

The methodology for assessing the IS has been described in detail in a previous publication^14^. Briefly, we processed two consecutive sections of formalin-fixed, paraffin-embedded tumor samples and applied immunohistochemical techniques to identify CD3+ and CD8 + T cells using specific rabbit monoclonal antibodies (2GV6 and SP57, respectively). Automated staining was performed on the Ventana BenchMark Ultra system, followed by visualization using the OptiView DAB kit (Roche Diagnostics, Germany). The CD3+ and CD8+ stained slides were digitized using an Aperio AT2 scanner (Leica Biosystems, Germany) at 40x magnification and exported in SVS format to the digital pathology software.

Using QuPath digital pathology software, the tumor invasive margin (IM) and tumor center (TC) were manually annotated by a trained physician, with a subset of these annotations validated in consultation with an immunopathology expert using open-source digital pathology software (QuPath v1.0.2)^30^. The IM was defined as a 1 mm wide perimeter at the invasive front of the tumor, and the TC was defined as the tumor mass consisting of adjacent CRC glands. The lymphocyte count per square millimeter was determined using fixed parameters with the automated cell detection function of the software. The IS was calculated from the percentile scores of CD3+ and CD8+ cell densities in both IM and TC areas, with the average of these four metrics (CD3 + /IM, CD3 + /TC, CD8 + /IM, and CD8 + /TC) determining the final IS. For statistical analysis purposes, the IS was categorized based on the validated optimal cut-off of 25th percentile as follows: IS-low (<25th percentile) and IS-high (≥25th percentile)^14^.

### Derivation of the combined C-reactive protein and immune cell score profile

To develop a novel prognostic biomarker profile for CRC patients, we combined the levels of poCRP and the IS into a unified classification termed the C-Reactive protein and Immune cell Score (CRIS) profile. CRIS categories were defined by integrating the dichotomized poCRP and IS variables into four categories as follows: CRIS-1 (poCRP-low + IS-high), CRIS-2 (poCRP-low + IS-low), CRIS-3 (poCRP-high + IS-high), and CRIS-4 (poCRP-high + IS-low).

### Outcomes

Endpoints assessed included overall survival (OS) and CRC-specific survival (CSS), defined as time to death from any cause or death due to CRC, respectively. In addition, we assessed relapse-free survival (RFS), defined as time to either recurrence of CRC or death attributable to CRC. Survival time was calculated in days from the date of blood collection to the occurrence of each endpoint. Follow-up ended on December 31, 2021, with censoring at the last recorded date on which patients were known to be alive.

### Statistical analyses

Descriptive statistics were used to summarize participant demographics and clinical characteristics, with continuous variables presented as medians with interquartile ranges (IQR) and categorical variables as counts and percentages. All analyses were conducted in R (version 4.3.3) with a two-sided significance threshold of *p* < 0.05. Missing data—excluding poCRP, IS status, event times, and survival endpoints—were addressed using multiple imputation via the “*mice*” package (version 3.17).

The correlation between continous poCRP and IS for the overall study population, as well as stratified by time since surgery and cancer stage at diagnosis, were assessed using the Pearson correlation analysis. The distribution of categories of poCRP and IS status by post-operative blood collection time was visualized with box plots, and their categorical correlation assessed via Cohen’s kappa. We assessed the dose-response associations between individual poCRP and IS with survival using restricted cubic splines. Kaplan-Meier plots (package “*survminer*” version 0.5) were used to visualize survival probabilities for the different CRIS profiles. Univariable and multivariable Cox regression models (adjusted for age, sex, TNM stage, tumor site/location, adjuvant chemotherapy, timing of blood sampling, and BMI) quantified individual and joint associations of poCRP and IS status with survival outcomes (package “*survival*” version 3.5−8). We also assessed the interaction between poCRP and IS in predicting survival by including a continuous-by-continuous interaction term (poCRP × IS) in the Cox regression models. Prognostic performance was measured using C-indices and Integrated Brier Scores to compare models with and without the CRIS profile (package “*compareC*” version 2.2−12).

Subgroup analyses were conducted based on blood sampling times (0.5–3, 3–6, 6–12, and >12 months post-surgery), and a sensitivity analysis was performed for patients with non-metastatic CRC. A secondary exploratory analysis in 405 patients who received adjuvant chemotherapy was conducted by stratifying them by timing of blood collection (before, during, and after chemotherapy) and assessing the CRIS profile’s prognostic value using multivariable Cox regression models. For CSS and RFS, Fine-Gray models accounted for competing risks (package “*cmprsk*” version 2.2−12), and for all time-to-event analyses, proportional hazards assumptions were checked by visualizing Schoenfeld residual plots.

## Supplementary information

Supplementary Materials

## Data Availability

Information on how to access the data can be found at http://dachs.dkfz.org/dachs/R codes used for the analyses can be can be found on GitHub (https://github.com/taffy-giff/CRIS-and-CRC-Prognosis) or requested from the corresponding author.

## Code availability

R codes used for the analyses can be can be found on GitHub (https://github.com/taffy-giff/CRIS-and-CRC-Prognosis) or requested from the corresponding author.
