# Supplementary material for: Predicting colorectal cancer survival by combined c-reactive protein and tumor immune score
Source: NPJ Precis Oncol. 2025 Nov 25;9:386. doi: 10.1038/s41698-025-01192-1 (PMC12663125; doi:10.1038/s41698-025-01192-1)
Supplement: Supplementary file 1 — Supplementary Materials [file 41698_2025_1192_MOESM1_ESM.docx]

**Supplementary Materials**

Contents

[**Supp. Fig 1** Patient selection flow diagram 2](#_Toc211088500)

[**Supp. Fig 2** Distribution of post-operative C-reactive protein and Immune cell score by time of blood collection after surgery 3](#_Toc211088501)

[**Supp. Fig 3** Correlation between post-operative C-reactive protein and immune cell score 4](#_Toc211088502)

[**Supp. Fig 4** Correlation between post-operative C-reactive protein and immune cell score stratified by time since surgery 5](#_Toc211088503)

[**Supp. Fig 5** Correlation between post-operative C-reactive protein and immune cell score stratified by cancer stage 6](#_Toc211088504)

[**Supp. Fig 6** CRIS status by CRC stage at diagnosis 7](#_Toc211088505)

[**Supp. Fig 7** Dose response associations of individual post-operative C-reactive protein (A) and Immune cell score (B) with survival outcomes 8](#_Toc211088506)

[**Supp. Fig 8** Kaplan-Meier plots for the CRC-specific (A) and recurrence-free (B) survival of patients by the CRIS profile. 9](#_Toc211088507)

[**Supp. Fig 9** Distribution of patients for the different blood sampling times in relation to receipt of adjuvant chemotherapy by stage (A), C-reactive protein (B) and Immune cell score (C). 10](#_Toc211088508)

[**Supp. Table 1** Association of categorical post-operative C-reactive protein and Immune cell score status in the study patients 11](#_Toc211088509)

[**Supp. Table 2** Cox regression analysis for the associations of elevated poCRP with survival outcomes by time of blood sampling 12](#_Toc211088510)

[**Supp. Table 3** Model performance of age and TNM stage with and without the CRIS profile for predicting survival 13](#_Toc211088511)

[**Supp. Table 4** Cox regression subgroup analysis by disease stage for the associations of CRIS profile with survival outcomes (stage I-III vs. IV) 14](#_Toc211088512)

[**Supp. Table 5** Cox regression sensitivity analysis for the associations of the CRIS profile with survival outcomes for patients with blood samples 2-weeks to 3-months post-surgery. 15](#_Toc211088513)

# **Supp. Fig 1** Patient selection flow diagram


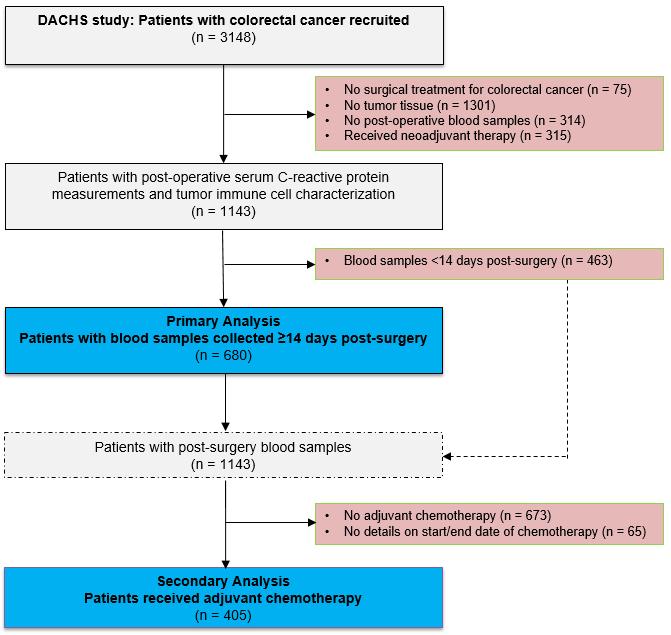


# **Supp. Fig 2** Distribution of post-operative C-reactive protein and Immune cell score by time of blood collection after surgery

| 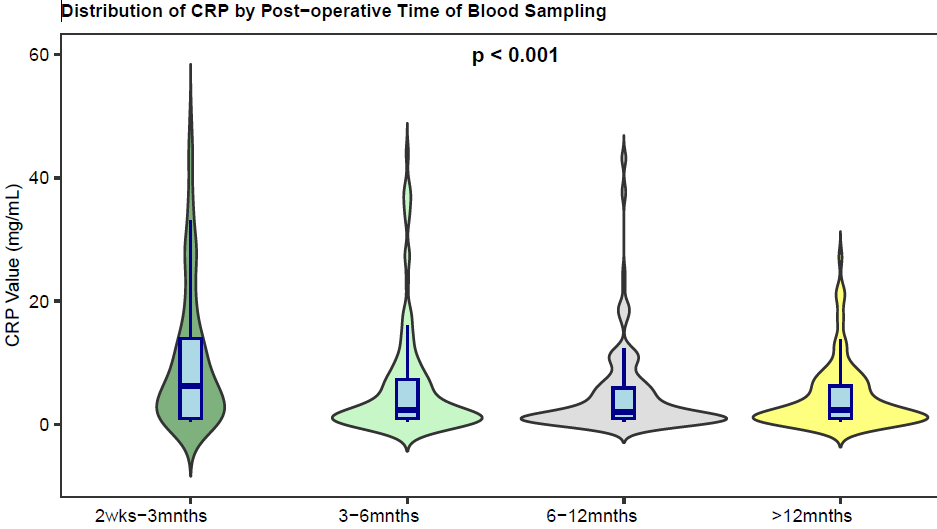 | 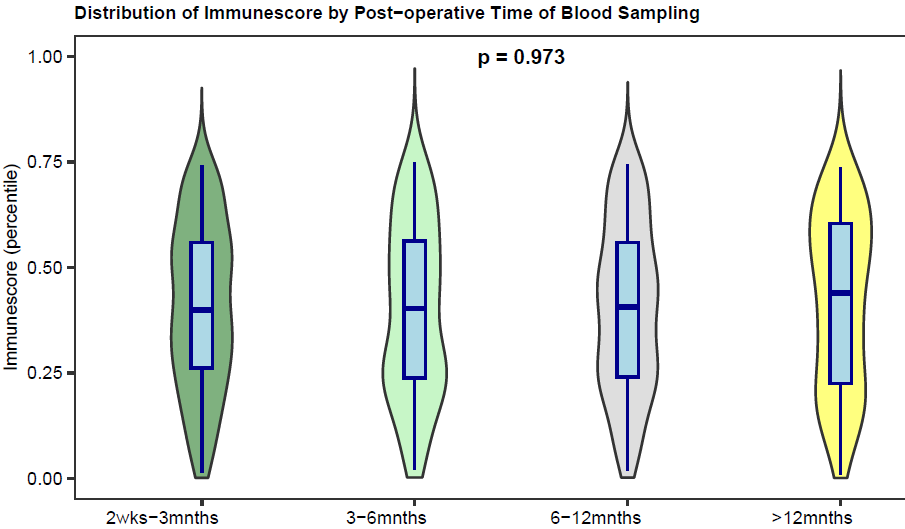 |
| --- | --- |

# **Supp. Fig 3** Correlation between post-operative C-reactive protein and immune cell score


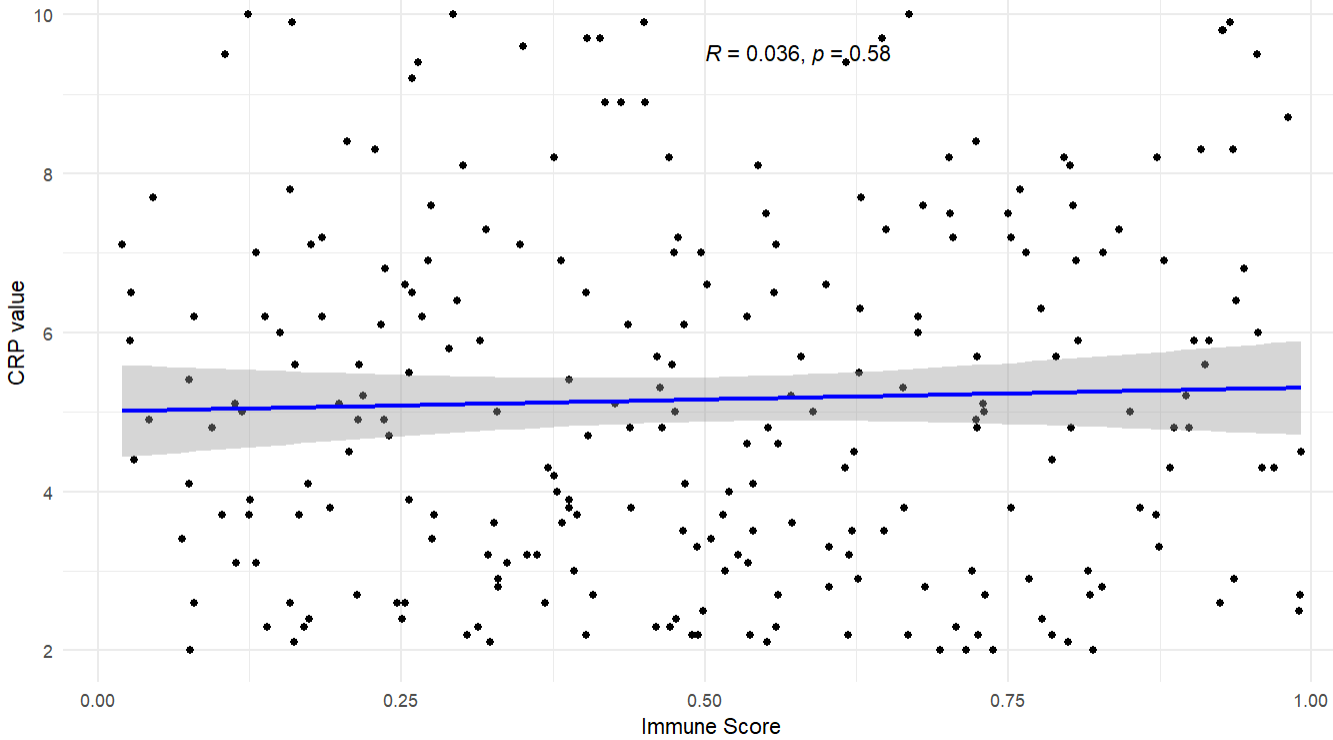


Notes: Unit of measurements are mg/L and mean percentiles for post-operative C-reactive protein and immune score, respectively

# **Supp. Fig 4** Correlation between post-operative C-reactive protein and immune cell score stratified by time since surgery

| 2 weeks – 3 months | 3 – 6 months |
| --- | --- |
| 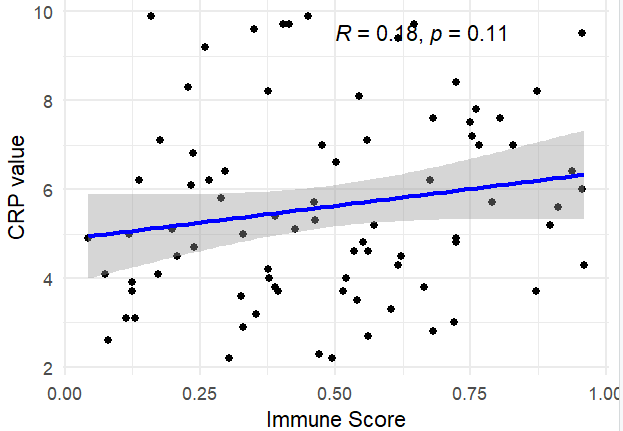 | 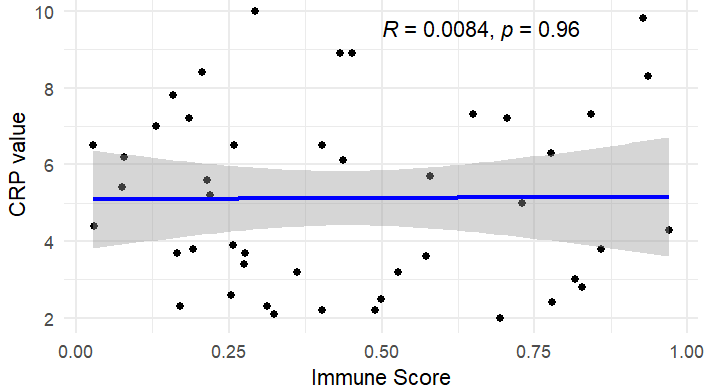 |
|  |  |
| 6 – 12 months | **>12 months** |
| 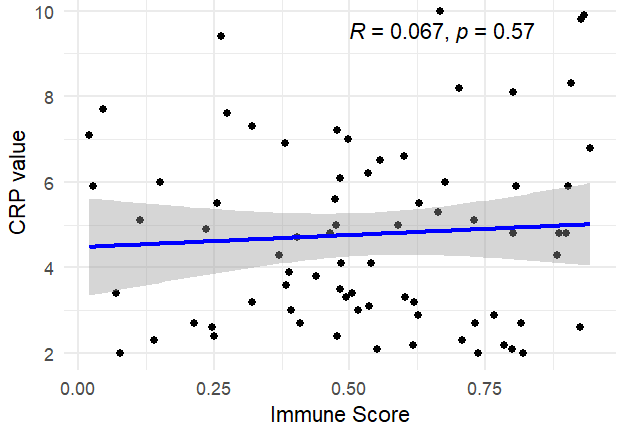 | 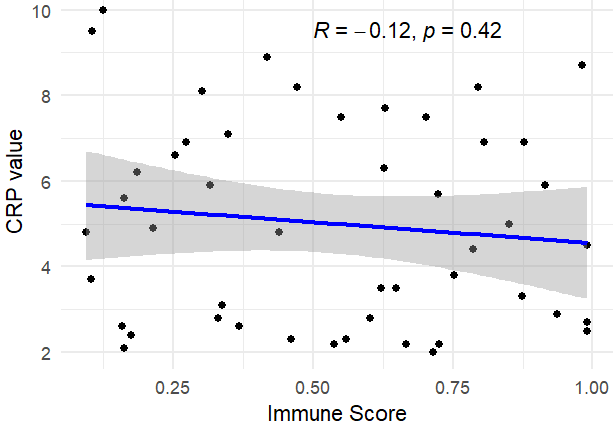 |

Notes: Unit of measurements are mg/L and mean percentiles for post-operative C-reactive protein and immune score, respectively

# **Supp. Fig 5** Correlation between post-operative C-reactive protein and immune cell score stratified by cancer stage

| Stage I | Stage II |
| --- | --- |
| 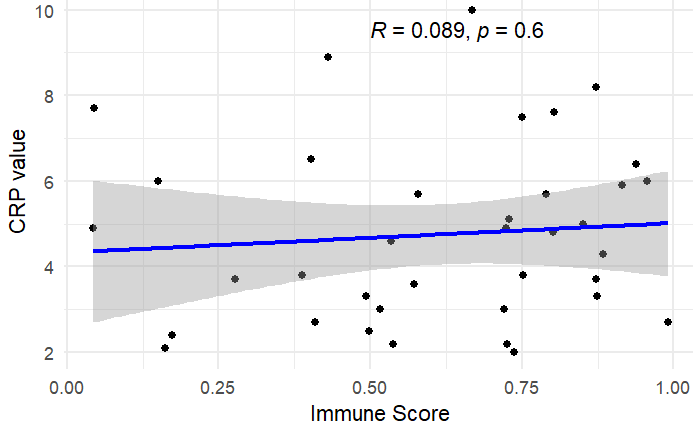 | 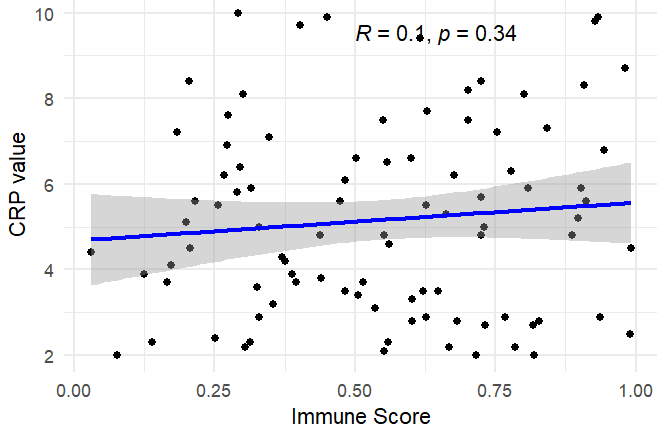 |
|  |  |
| Stage III | **Stage IV** |
| 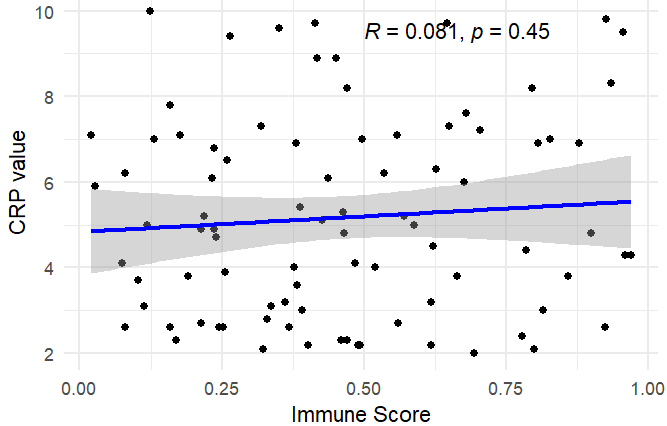 | 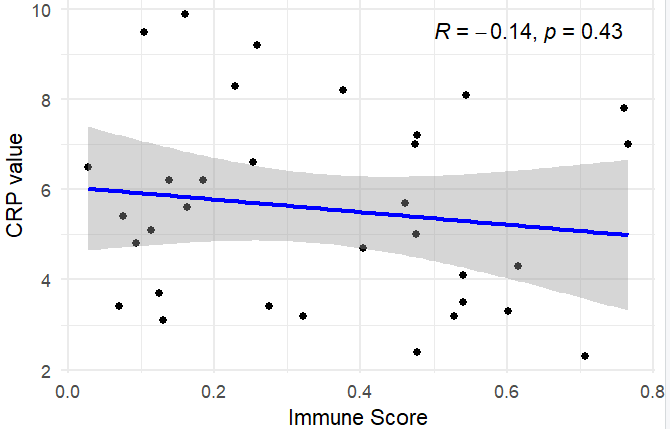 |

Notes: Unit of measurements are mg/L and mean percentiles for post-operative C-reactive protein and immune score, respectively

# **Supp. Fig 6** CRIS status by CRC stage at diagnosis

**
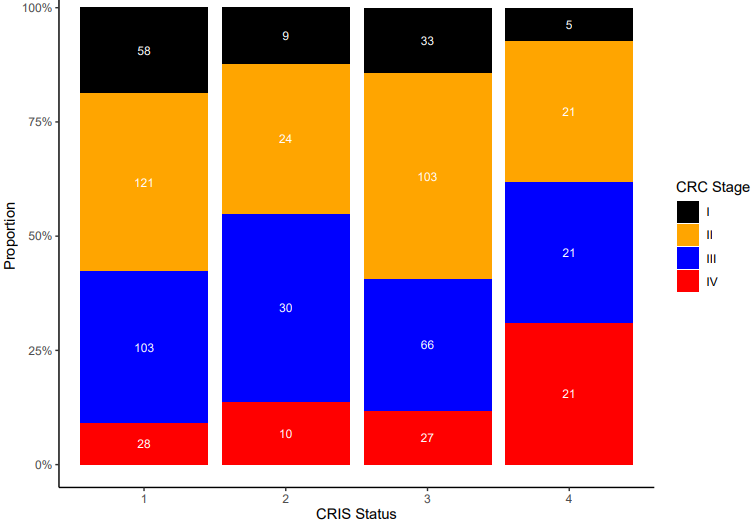
**

# **Supp. Fig 7** Dose response associations of individual post-operative C-reactive protein (A) and Immune cell score (B) with survival outcomes

|  | Overall Survival | CRC-Specific Survival | Relapse-Free Survival |
| --- | --- | --- | --- |
| A | 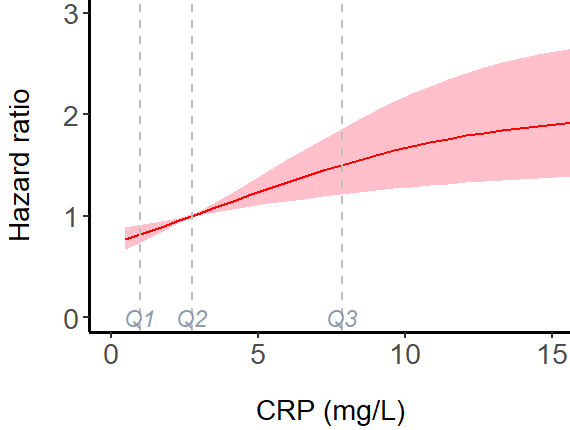 | 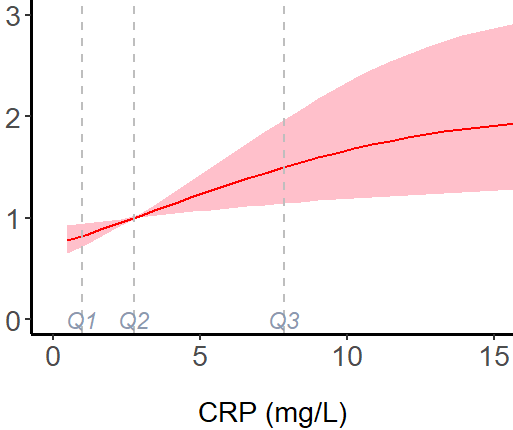 | 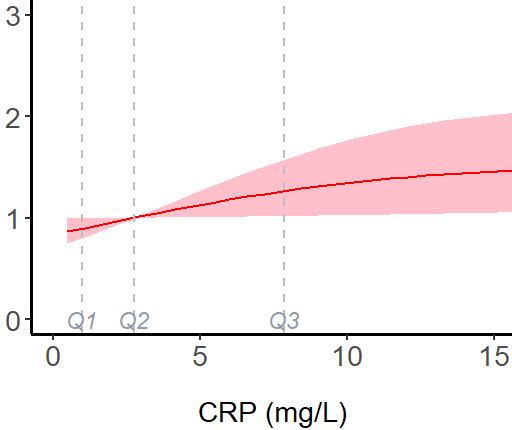 |
|  |  |  |  |
| B | 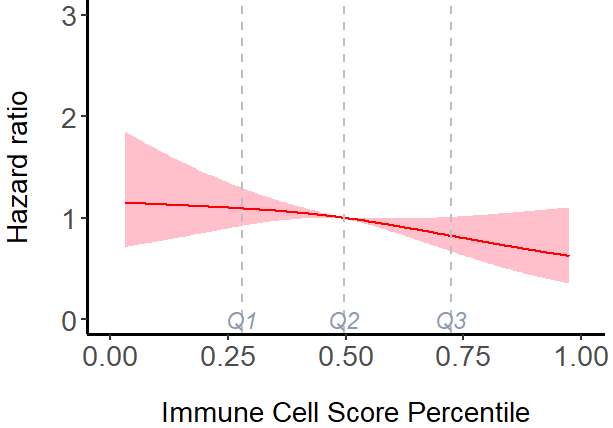 | 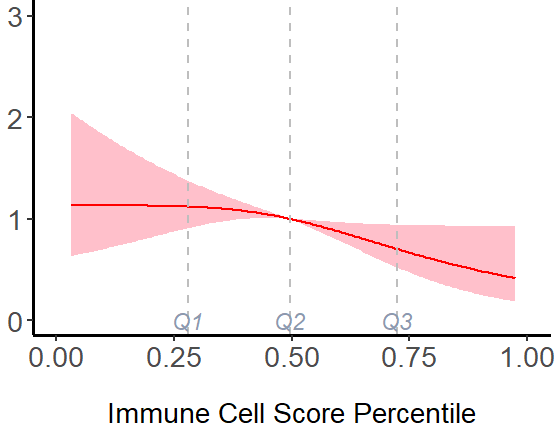 | 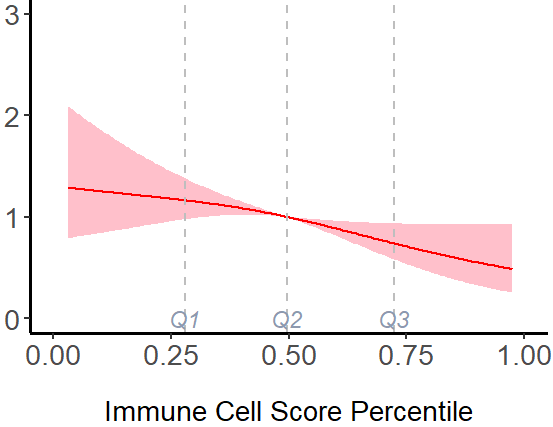 |

# **Supp. Fig 8** Kaplan-Meier plots for the CRC-specific (A) and recurrence-free (B) survival of patients by the CRIS profile.

| 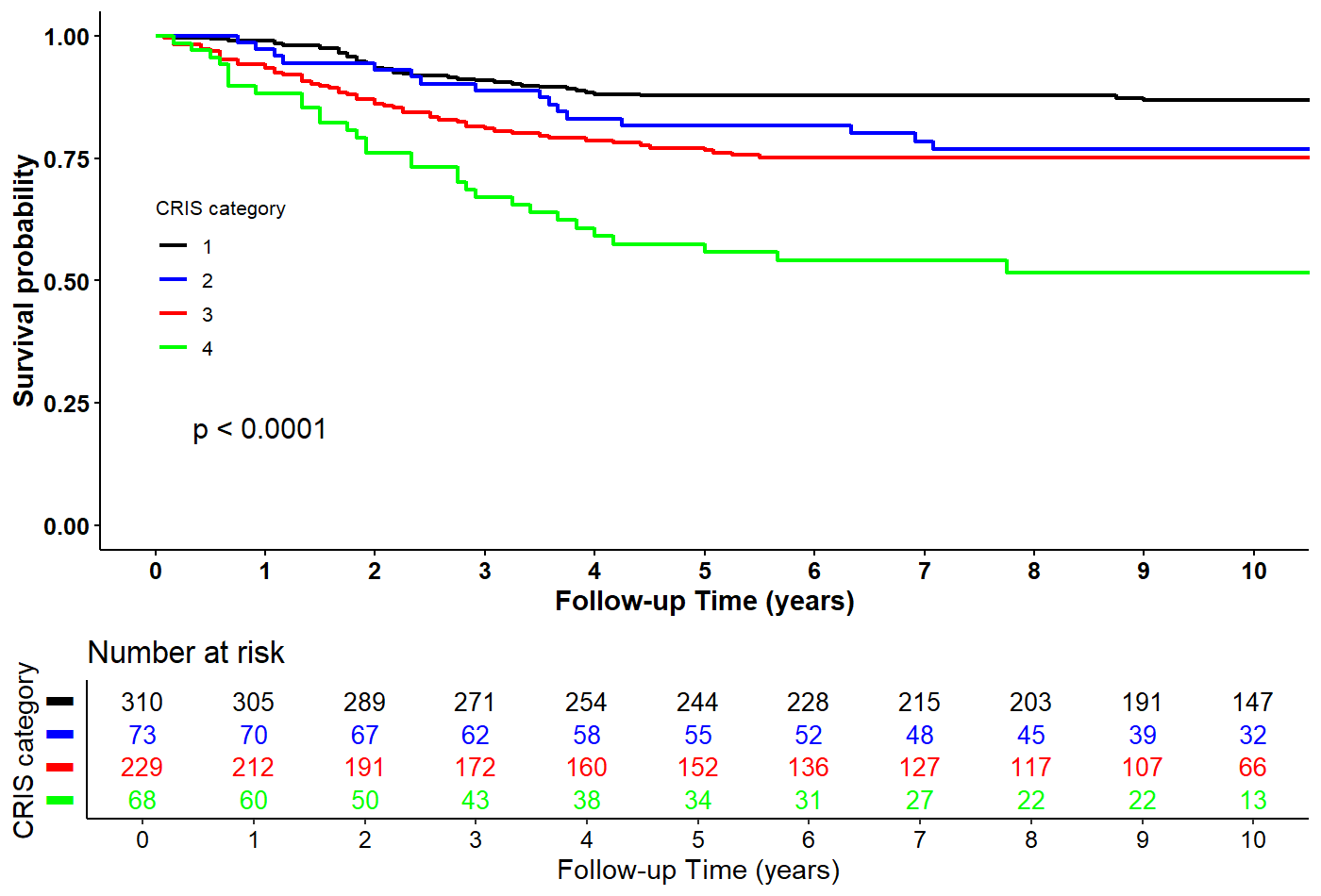  A | 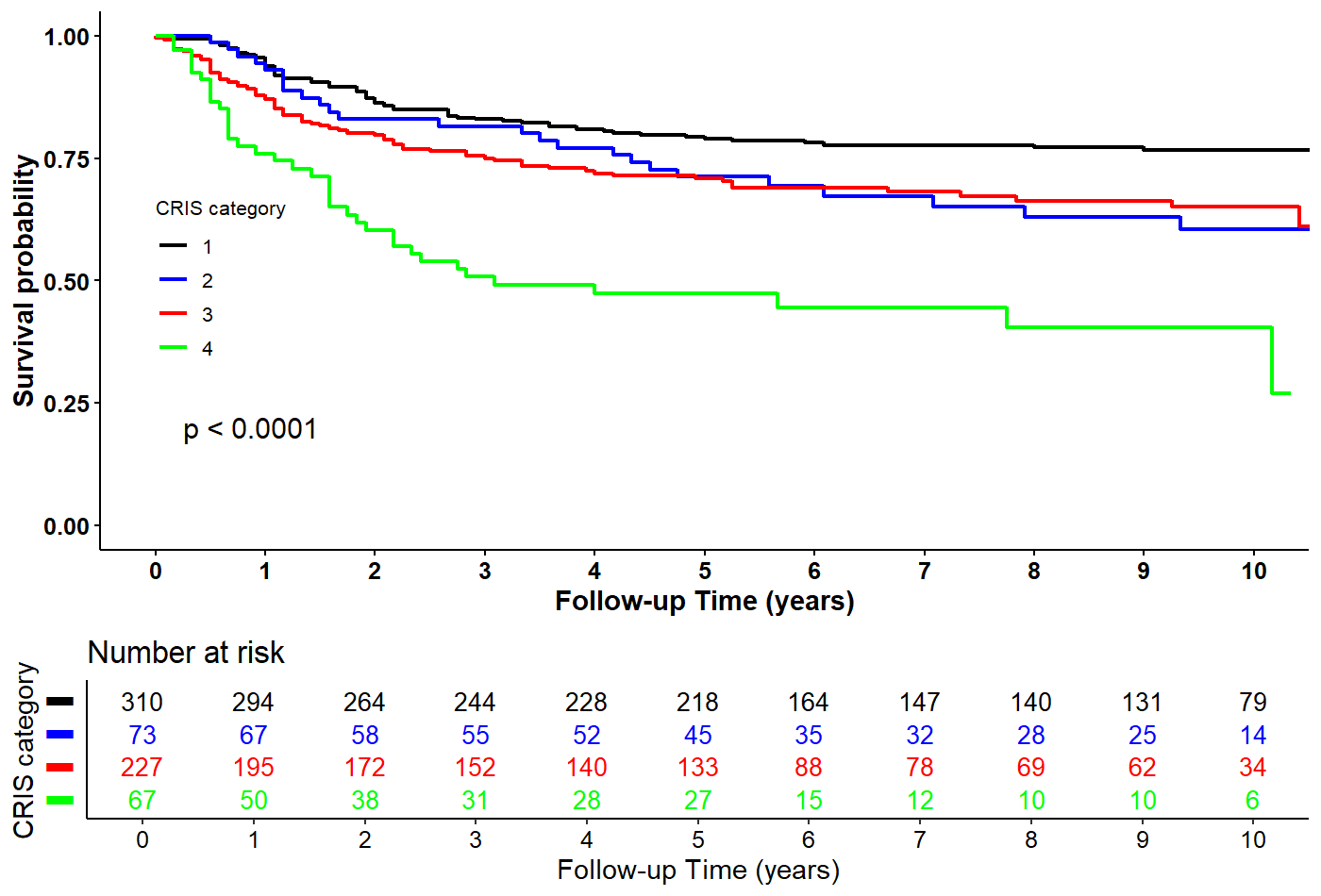  B |
| --- | --- |

# **Supp. Fig 9** Distribution of patients for the different blood sampling times in relation to receipt of adjuvant chemotherapy by stage (A), C-reactive protein (B) and Immune cell score (C).

| **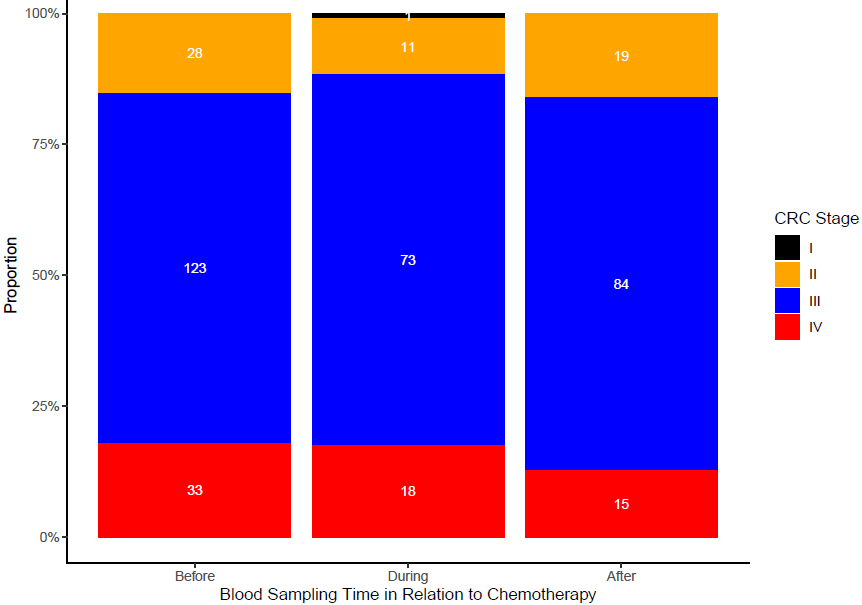**  Abbreviations: CRC, colorectal cancer; CRP, C-reactive protein | **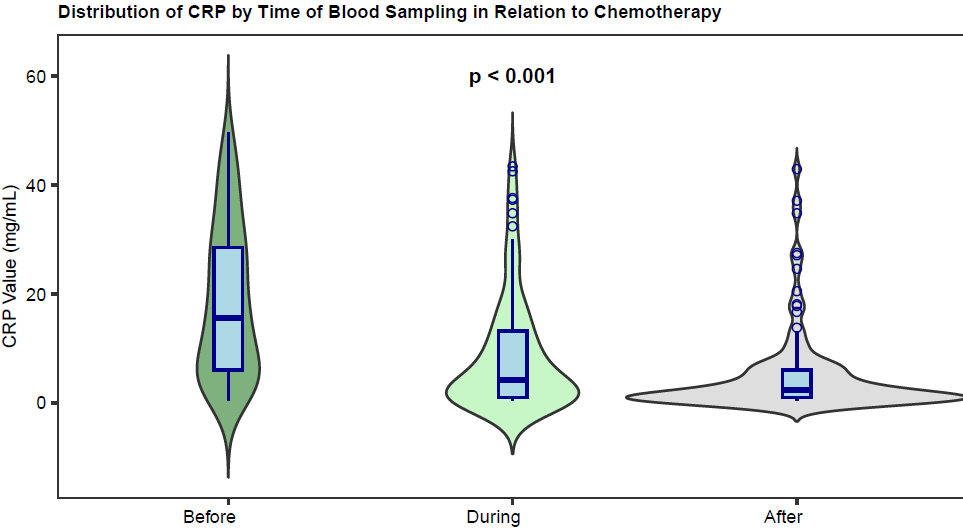**  A  B |
| --- | --- |
|  | **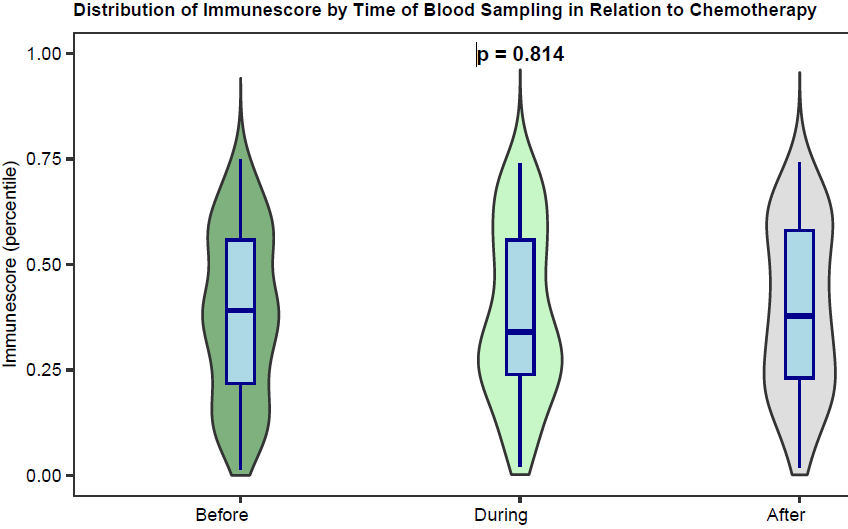**  C |

# **Supp. Table 1** Association of categorical post-operative C-reactive protein and Immune cell score status in the study patients

| **C-reactive protein** |  | Immunescore | |
| --- | --- | --- | --- |
|  |  | **Low** | **High** |
|  | **Low** | 73 (10.7%) | 310 (45.6%) |
|  | **High** | 68 (10.0%) | 229 (33.7%) |
|  | Cohen`s kappa coefficient: -0.04 (*p = 0.22*) | | |

# **Supp. Table 2** Cox regression analysis for the associations of elevated poCRP with survival outcomes by time of blood sampling

| **Prognostic Outcome** | **Time of Blood Sampling** | **Event Rate^1^** | **Model 1** (HR, 95% CI) | **Model 2** (HR, 95% CI) |
| --- | --- | --- | --- | --- |
| Overall Survival | 2 weeks – 3 months | 43% | **1·88 (1·20–2·96)** | 1·53 (0·96–2·43) |
|  | 3 – 6 months | 29% | 1·14 (0·58–2·24) | 1·19 (0·58–2·41) |
|  | 6 – 12 months | 35% | **1·98 (1·14–3·46)** | **1·92 (1·10–3·36)** |
|  | >12 months | 54% | **3·12 (2·10–5·10)** | **2·77 (1·35–5·65)** |
|  |  |  |  |  |
| CRC Specific Survival* | 2 weeks – 3 months | 32% | **2·34 (1·31–4·19)** | **1·83 (1·01–3·34)** |
|  | 3 – 6 months | 23% | 1·56 (0·69–3·54) | 1.47 (0·60–3·58) |
|  | 6 – 12 months | 19% | 1·54 (0·74–3·20) | 1·49 (0·71–3·11) |
|  | >12 months | 40% | **3·66 (1·62–8·25)** | **4·35 (1·87–10·15)** |
|  |  |  |  |  |
| Relapse-Free Survival* | 2 weeks – 3 months | 38% | **2·10 (1·28–3·43)** | **1·87 (1·13–3·10)** |
|  | 3 – 6 months | 31% | 1·35 (0·69–2·66) | 1·41 (0·70–2·85) |
|  | 6 – 12 months | 28% | 1·45 (0·82–2·57) | 1·53 (0·86–2·72) |
|  | >12 months | 46% | **1·94 (1·01–3·72)** | 1·88 (0·97–3·63) |
|  |  |  |  |  |

*Competing risk models were applied accounting for non-CRC deaths as a competing risk using the Fine-Gray model

^1^Calculated as a proportion of events/number of patients in the poCRP high group

Notes: Model 1 was univariable; Model 2 was adjusted for age + sex + TNM stage (low poCRP as the reference group); Bold numbers show statistically significant associations.

Abbreviations: CI, confidence interval; CRC, colorectal cancer; poCRP; post-operative C-reactive protein; HR, hazard ratio.

# **Supp. Table 3** Model performance of age and TNM stage with and without the CRIS profile for predicting survival

| **Prognostic outcome** | **Performance Metric** | **Model** | | |
| --- | --- | --- | --- | --- |
|  |  | Age + TNM stage | Age + TNM stage + CRIS status | *P-value* |
| Overall survival | C-index (95% CI) | 0·74 (0·70–0·77) | 0·75 (0·72–0·78) | *0·016* |
|  | Integrated Brier Score  (Percentage Change, 95% CI) | Reference | -0.50 (-1.00, -0.10) | *0.037* |
|  |  |  |  |  |
| CRC-specific survival | C-index (95% CI) | 0·82 (0·79–0·86) | 0·84 (0·81–0·87) | *0·049* |
|  | Integrated Brier Score  (Percentage Change, 95% CI) | Reference | -0.50 (-1.00, -0.10) | *0.013* |
|  |  |  |  |  |
| Relapse-free survival | C-index (95% CI) | 0·74 (0·71–0·78) | 0·76 (0·72–0·79) | *0·014* |
|  | Integrated Brier Score  (Percentage Change, 95% CI) | Reference | -0.50 (-1.00, -0.10) | *0.039* |

Abbreviations: 95% CI, 95% confidence interval; CRIS, **C**-**R**eactive protein and **I**mmune cell **S**core profile; TNM, tumor-node-metastasis.

# **Supp. Table 4** Cox regression subgroup analysis by disease stage for the associations of CRIS profile with survival outcomes (stage I-III vs. IV)

| **Prognostic Outcome** | **CRIS category** | **Stage III** (HR, 95% CI) | **Stage IV** (HR, 95% CI) |
| --- | --- | --- | --- |
| Overall Survival | 1 | 1·00 (ref) | 1·00 (ref) |
|  | 2 | 1·18 (0·65–2·13) | 1·50 (0·61–3·72) |
|  | 3 | **1·65 (1·13–2·40)** | **3·10 (1·66–5·80)** |
|  | 4 | **2·45 (1·43–4·21)** | **2·14 (1·11–4·11)** |
| *P-trend* |  | *<0.001* | *0.004* |
| CRC Specific Survival* | 1 | 1·00 (ref) | 1·00 (ref) |
|  | 2 | 1·69 (0·74–3·83) | 1·58 (0·64–3·92) |
|  | 3 | **2·30 (1·30–4·05)** | **2·92 (1·55–5·52)** |
|  | 4 | **4·49 (2·27–8·91)** | 1·90 (0·96–3·75) |
| *P-trend* |  | *<0.001* | *0.014* |
| Relapse-Free Survival* | 1 | 1·00 (ref) | 1·00 (ref) |
|  | 2 | 1·58 (0·89–2·80) | 1·63 (0·70–3·81) |
|  | 3 | **1·60 (1·06–2·41)** | **2·27 (1·25–4·15)** |
|  | 4 | **3·07 (1·78–5·31)** | 1·85 (0·98–3·51) |
| *P-trend* |  | *<0.001* | *0.022* |
|  |  |  |  |

*Models for CSS and RFS predictions accounted for non-CRC death as a competing risk using the Fine-Gray model

Notes: Multivariable model was adjusted for sex + age + adjuvant chemotherapy + time of blood sampling; Bold numbers show statistically significant associations. P-trend was based on modelling CRIS as a numeric variable.

Abbreviations: CI, confidence interval; CRC, colorectal cancer; HR, hazard ratio; ref, reference.

# **Supp. Table 5** Cox regression sensitivity analysis for the associations of the CRIS profile with survival outcomes for patients with blood samples 2-weeks to 3-months post-surgery.

| **Prognostic Outcome** | **CRIS category** | **Model 1** (HR, 95% CI) | **Model 2** (HR, 95% CI) |
| --- | --- | --- | --- |
| Overall Survival | 1 | 1·00 (ref) | 1·00 (ref) |
|  | 2 | 1·50 (0·60–3·72) | 0·74 (0·29–1·88) |
|  | 3 | **1·96 (1·16–3·29)** | 1·47 (0·87–2·50) |
|  | 4 | **2·35 (1·21–4·53)** | 1·24 (0·63–2·45) |
|  |  |  |  |
| CRC Specific Survival* | 1 | 1·00 (ref) | 1·00 (ref) |
|  | 2 | 2·49 (0·85–7·30) | 0·97 (0·33–2·91) |
|  | 3 | **2·63 (1·30–5·33)** | 1·83 (0·88–3·79) |
|  | 4 | **4·03 (1·79–9·07)** | 1·78 (0·76–4·16) |
|  |  |  |  |
| Relapse-Free Survival* | 1 | 1·00 (ref) | 1·00 (ref) |
|  | 2 | **2·54 (1·04–6·24)** | 1·41 (0·56–3·52) |
|  | 3 | **2·22 (1·23–4·02)** | **1·92 (1·04–3·54)** |
|  | 4 | **4·24 (2·13–8·43)** | **2·57 (1·25–5·28)** |
|  |  |  |  |

*Models for CSS and RFS predictions accounted for non-CRC death as a competing risk using the Fine-Gray model

^1^Calculated as a proportion of events/number of patients in the poCRP high group

Model 1 was univariable; Model 2 was adjusted for age + sex + TNM stage.

Abbreviations: CI, confidence interval; CRC, colorectal cancer; HR, hazard ratio; ref, reference.
